# Uncovering Hidden Genetic Diversity in Ancient Palestinian Olive Rootstocks and Insights into Levantine Domestication

**DOI:** 10.3390/plants15182838

**Published:** 2026-09-16

**Authors:** Ahmad Alomary, Roberto Mariotti, Osama Alabdallah, Lorenzo Cruciani, Muhammad Ajmal Bashir, Mohammed M. Abueid, Raed Alkowni, Silvia Casciani, Soraya Mousavi

**Affiliations:** 1 Environmental Sciences Program, An-Najah National University, Nablus P.O. Box 7, Palestine; a.alomary1@gmail.com; 2 National Research Council, Institute of Biosciences and Bioresources, 06128 Perugia, Italy; roberto.mariotti@cnr.it (R.M.); lorenzocruciani@regione.umbria.it (L.C.); muhammadajmalbashir@cnr.it (M.A.B.); silviacasciani@cnr.it (S.C.); 3 National Agriculture Research Center (NARC), Ministry of Agriculture, Jenin P.O. Box 209, Palestine; oalabdallah@gmail.com (O.A.); m_abueid@yahoo.com (M.M.A.); 4 Department of Chemistry and Biology, Faculty of Science, An-Najah National University, Nablus P.O. Box 7, Palestine; ralkowni@najah.edu

**Keywords:** *Olea europaea* L., genetic diversity, ancient rootstocks, SSR markers, olive domestication, historical grafting

## Abstract

The Levant is a primary cradle of olive (*Olea europaea* L.) domestication. While canopy diversity is well-documented, historical grafting obscures an untapped genetic reservoir within ancient rootstocks. This study utilized a dual-sampling approach, genotyping 142 canopy and rootstock samples across the West Bank using 10 SSR loci, benchmarked against 695 global cultivars. Molecular analysis uncovered 41 distinct grafting events among 49 paired trees, indicating a historical grafting practice where ‘Baladi’ canopies were combined with 45 unique rootstock genotypes and in five cases with the ‘Safrawi’ cultivar. Parentage analysis suggested that these rootstocks formed a putatively interbreeding population. The eastern founder ‘Safrawi’ was identified as candidate parent for several rootstocks, whereas ‘Baladi’ scions remained genetically distinct. Bayesian structure analysis (K = 3) showed the West Bank population belongs predominantly to the ancestral Eastern Mediterranean pool (POP3), remaining isolated from central and western breeding loops. The local germplasm exhibited high allelic richness and observed heterozygosity (*Ho* up to 0.97), rivaling global collections. These findings provide direct molecular evidence of historical agricultural practices, establishing the West Bank as an important reservoir of the Eastern Mediterranean gene pool. These putative resilient genotypes could offer valuable traits for future breeding programs, making their conservation a priority.

## 1. Introduction

The olive (*Olea europaea* L.) is one of the earliest domesticated fruit trees in the Mediterranean Basin and a foundational element of its agricultural and cultural history [1]. The Levant, a region which includes modern-day Palestine alongside neighboring countries, is widely recognized as the primary cradle of olive domestication [2]. Archaeological and archaeobotanical evidence, such as the exceptionally well-preserved olive stones and oil production facilities found at submerged sites off the Carmel Coast (e.g., Kfar Samir), indicates that organized olive oil production and early domestication processes were already underway by the late Neolithic and Chalcolithic periods, approximately 6500 to 7000 years ago [3,4]. Cultivation further intensified during the Early Bronze Age; significant increases in olive pollen and the discovery of oil extraction installations and crushed olive stones at sites like Tel Yarmouth reveal the emergence of a highly organized, export-driven olive oil industry [4,5]. In Palestine, the olive tree transcends routine agricultural production; it serves as a primary cultural symbol of resilience, heritage, and identity, dominating regional agro-ecosystems and underpinning local rural economies across the West Bank and Gaza [6].

Millennia of continuous cultivation in the diverse and often challenging environmental conditions of the region have fostered a rich genetic reservoir within local olive landraces and monumental trees, locally referred to as “Roumi” [7]. These ancient, living trees function as critical in situ repositories of genetic diversity, preserving traits that confer resilience to environmental stressors like drought, poor soils, and diseases. Previous studies on the primary Palestinian cultivars, such as Nabali Baladi, Nabali Mohassan, and Souri, have demonstrated substantial intra-varietal genetic diversity, which has been shaped by centuries of vegetative propagation, somatic mutations, and agricultural selection [8,9].

However, despite the diversity observed in the canopies of these ancient trees, historical grafting practices have often obscured an even deeper and older genetic pool hidden within their rootstocks [10,11]. Grafting has been intimately linked to olive cultivation in the Levant since antiquity to propagate desirable clones that do not easily root and to increase overall tree survival [12]. Traditional farmers frequently utilized wild saplings (oleasters), feral seedlings, or specifically selected primeval clones as rootstocks [11]. Because superior canopy clones, such as the widespread Souri, were selectively propagated over vast areas, the highest genetic variation is preserved within the rootstocks of these grafted monumental trees [13]. Direct paired comparisons of canopies and rootstocks from the same ancient trees have historically been lacking in Palestinian surveys, yet this dual-sampling approach is essential to uncover “hidden” paleo-varieties representing remnants from the earliest phases of olive cultivation [12].

Understanding the hidden rootstock diversity in Palestine also provides a crucial window into the broader phylogeography and genetic relationships of the olive tree across the Middle East and the Mediterranean Basin [14]. Genetic analyses of maternally inherited chloroplast DNA reveal that the Eastern Mediterranean (the Levant) is the primary source of the dominant E1 lineage, which subsequently spread westward across the Mediterranean alongside human migrations and trade [1]. The Levantine gene pool has contributed heavily to the establishment of the contemporary olive germplasm in countries such as Cyprus, Lebanon, Greece, Italy, and Spain [10,14]. Furthermore, recent research has uncovered an unexpected eastward route of genetic differentiation, illustrating that ancient Levantine and Mesopotamian olive lineages also migrated toward the Iranian plateau long before full domestication occurred [15].

By profiling both canopy and rootstock tissues from the same ancient trees in the West Bank, this study aims to access the uncharacterized reservoir of genetic diversity hidden beneath the soil [12]. Benchmarking these paired genotypes against standardized global panels will not only quantify the distinctiveness of Palestinian monumental olives but also clarify their ancestral and genetic affinities to other populations throughout the Middle East and the Mediterranean Basin.

## 2. Results

### 2.1. Field Characterization of Ancient Olive Trees

**Figure 1:**
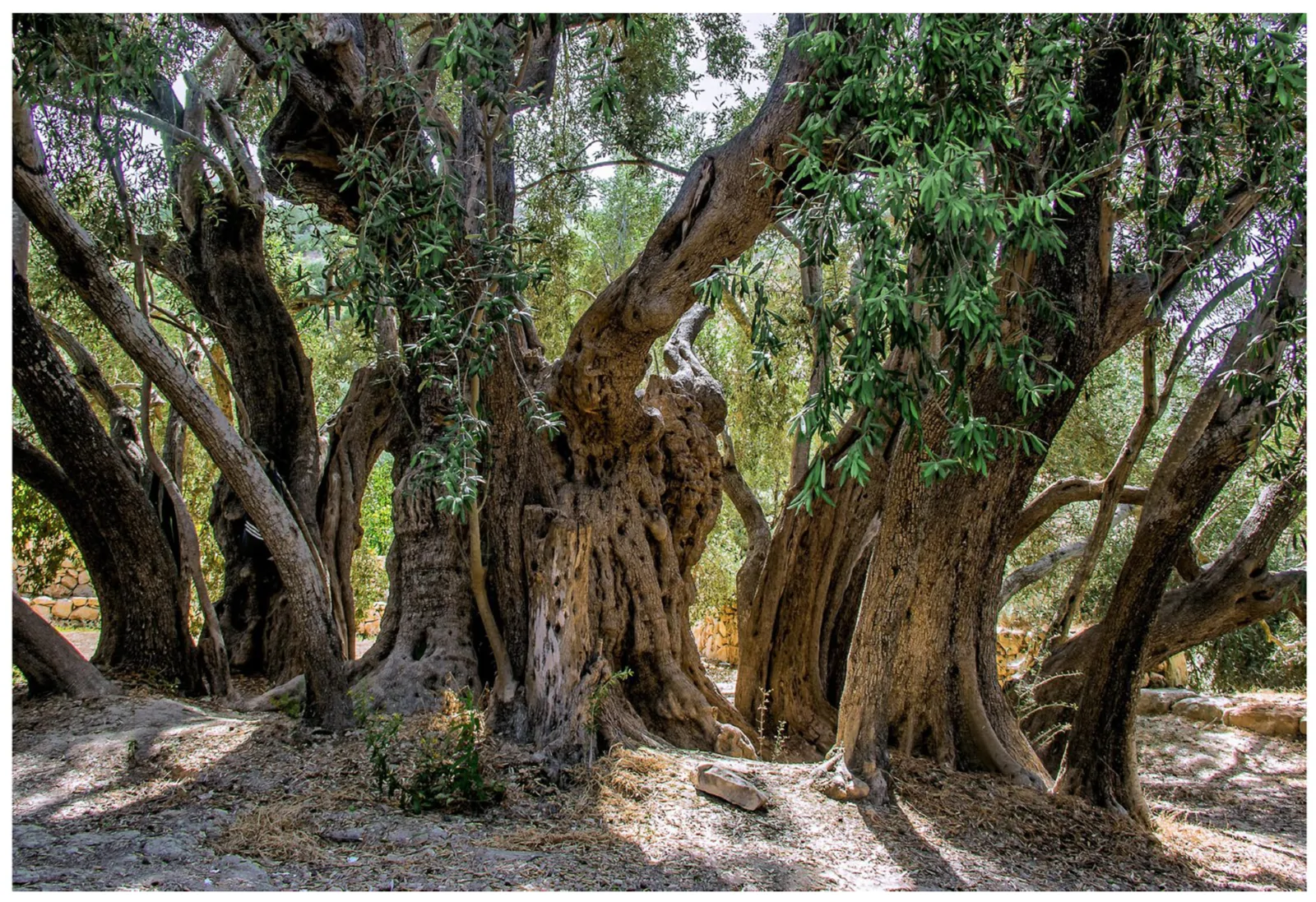
Sample 94_094BWAL, featuring a trunk diameter of approximately 6.02 m, represents a unique genotype.

A field survey was conducted across five governorates in the West Bank, encompassing regions such as Jenin (including Rummana, Yamon, Burqin, and Qabatiya), Tubas, Nablus, Hebron, and Bethlehem (Walaja). In total, 93 olive trees were assessed, including 54 monumental trees with the presumed age of more than 100 years old. These trees were in irregular groves, urban or peri-urban edges, and semi-natural habitats characterized by low-input management. Soil management practices varied across the sites: most fields were regularly tilled, some retained spontaneous ground cover with minimal intervention, and others combined tillage with legume cover crops. Currently, these valuable genetic resources face ongoing risks from urbanization, agricultural abandonment, fire, and uprooting. The surveyed trees exhibited diverse trunk structures, with 78 classified as mono-cormic (single-stemmed) and the remaining 15 identified as poly-cormic (possessing two or more stems). Trunk diameters (mean 1.49 m) ranged from 0.80 m to a maximum of 6.02 m, a peak exemplified by the poly-cormic tree ‘94C’ in Bethlehem-Walaja (Figure 1).

### 2.2. Genetic Diversity Characterized According to SSR Markers

**Figure 2:**
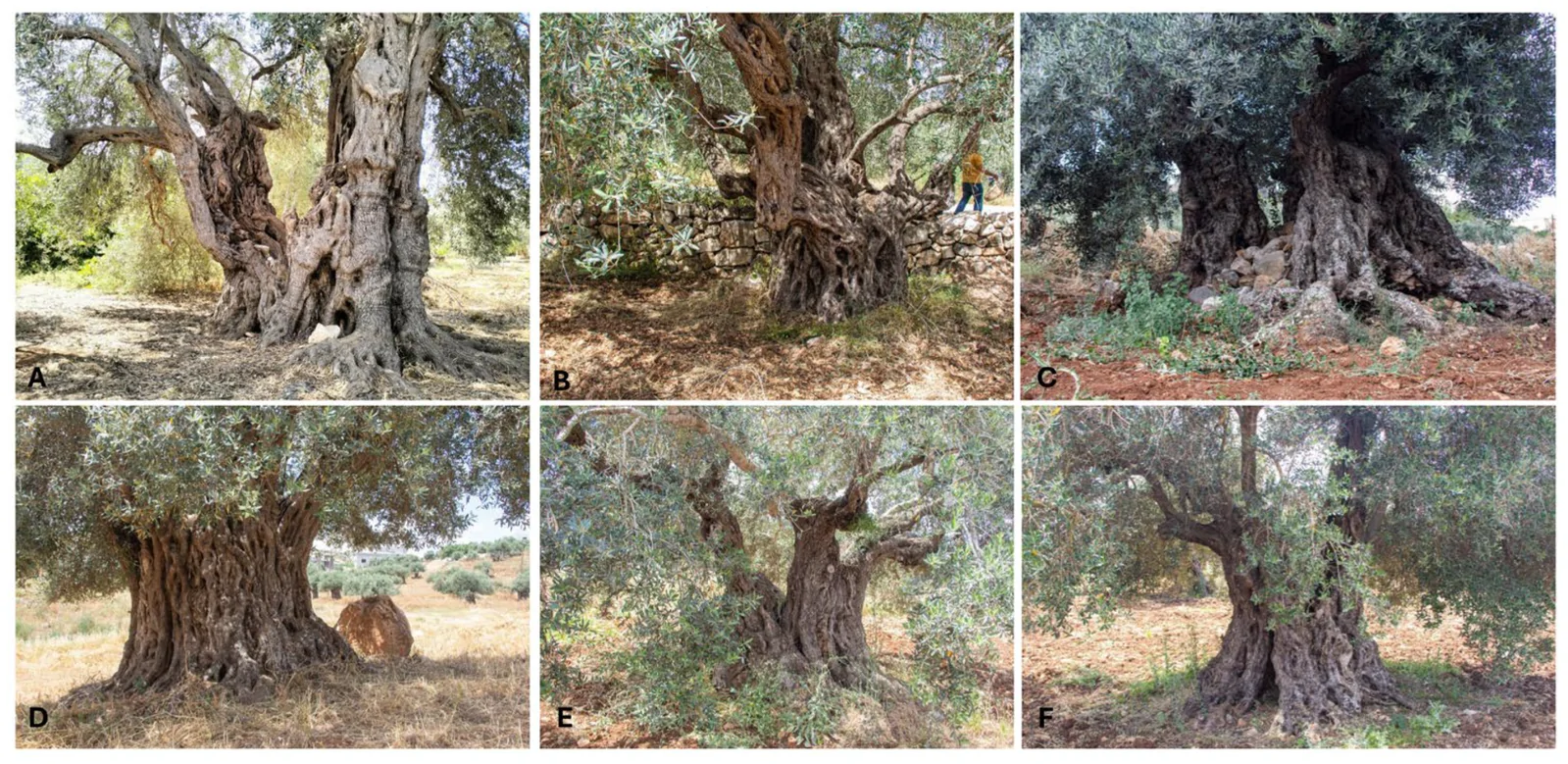
Canopy/rootstock genetic identity of ancient Palestinian olive trees. (**A**–**D**) Cv. Baladi grafted on a unique genotype; (**E**) a unique genotype grafted on another unique genotype; (**F**) Cv. Baladi grafted on Cv. Safrawi.

Molecular characterization of the 142 collected leaf samples (92 canopy and 50 rootstock) identified 79 samples as cv. ‘Baladi’, five as ‘Safrawi’, and two as ‘Frantoio’. The remaining 56 samples possessed previously uncharacterized genotypes, representing 48 distinct, unique genetic profiles (considering also three cultivars Baladi, Safrawi and Frantoio). Among the 49 paired trees, 41 grafting events were detected where the scion and rootstock possessed different genetic identities (Figure 2). The most frequent grafting combination involved a ‘Baladi’ canopy grafted onto an uncharacterized rootstock genotype, accounting for 34 of these events. Additionally, five trees featured a ‘Baladi’ canopy grafted onto a ‘Safrawi’ rootstock. One reverse event was documented (tree 27 from Tubas-Tayasir), where a unique genotype canopy was found grafted onto a ‘Baladi’ rootstock. These results indicate a historical grafting practice where uniform clonal scions, predominantly cv. ‘Baladi’, were grafted onto genetically diverse rootstock genotypes (Table S1). To avoid clonal bias in genetic distance and multivariate analyses, clonal redundant profiles within the 142 newly genotyped Palestinian samples were removed, leaving 48 unique genotypes.

**Figure 3:**
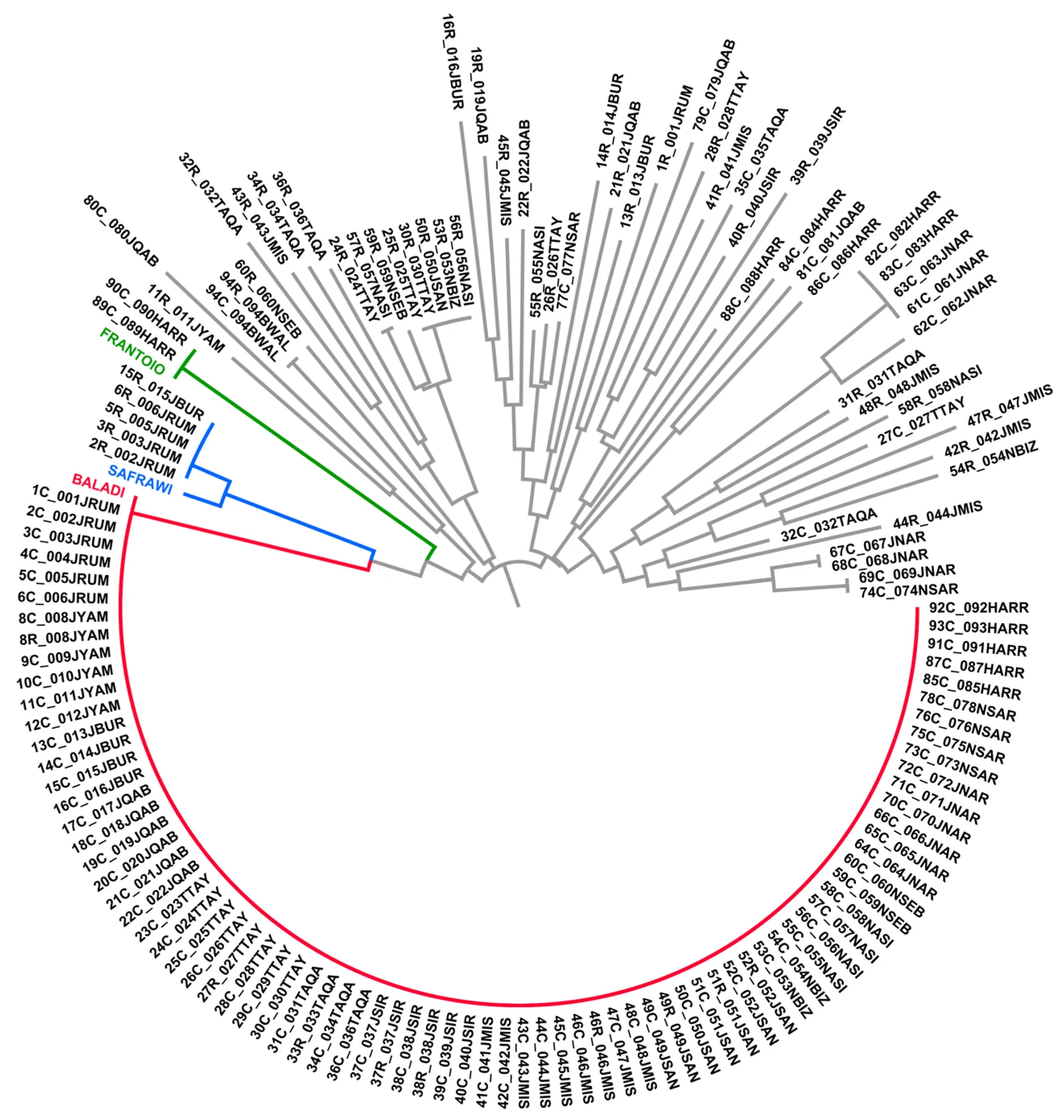
Dendrogram showing the Neighbor-Joining genetic distance, depicting genetic relationships of 142 Palestinian samples. Red branches indicate all genotypes that are clones of the cultivar ‘Baladi’; blue branches show clones of the cultivar ‘Safrawi’; green branches illustrate clones of the cultivar ‘Frantoio’; and grey branches show unique, unknown genotypes.

The Neighbor-Joining (NJ) genetic-distance dendrogram of the West Bank samples illustrates a strong clustering of the canopy samples, reflecting their dominant and shared ‘Baladi’ genetic identity. In contrast, the rootstock samples (indicated with the suffix ‘R’, such as 24R_024TTAY, 31R_031TAQA, 45R_045JMIS, and 57R_057NASI) do not cluster together but are widely distributed across different clades. This broad dispersion corresponds to the high number of unique genotypes discovered in Table S1 and visually underscores the substantial genetic variation preserved within the root systems (Figure 3).

To contextualize this diversity globally, a second NJ dendrogram benchmarked the 48 unique West Bank genotypes against an extensive database of 695 world cultivars, yielding a comparative dataset of 743 distinct genotypes (Figure S1). The ancient West Bank olives are positioned at a considerable genetic distance from widespread commercial cultivars such as Arbequina, Koroneiki, Arbosana, Frantoio and Coratina. The segregation of these ancient Palestinian rootstocks highlights them as an isolated, localized gene pool containing rare paleo-genotypes largely unrepresented in standard Mediterranean and global breeding collections (Figure S1). To complement the hierarchical NJ clustering and represent genetic relationships non-hierarchically, a Principal Coordinates Analysis (PCoA) was performed on the comparative database of 743 genotypes (Figure S2). The first three coordinate axes explained 8.31%, 4.64%, and 4.52% of the genetic variation, respectively, with Axes 1 and 2 explaining a cumulative 12.95% of the variance. Axis 1 separates the western Mediterranean pool on the negative coordinate space from the central and eastern germplasms on the positive coordinate space. The PCoA projection shows the ‘Baladi’ canopy samples group in a confined, overlapping space, while the unique Palestinian rootstocks are distributed across the Eastern Mediterranean coordinate quadrant, with several individuals projecting towards the central Mediterranean space.

A detailed quantitative assessment of genetic diversity for 743 West Bank and international reference cultivars was performed using 10 SSR loci (Table 1).

The West Bank population displayed high allelic richness, yielding a total of 131 alleles across the 10 loci, compared to 257 alleles found in the broader World germplasm reference. Within the West Bank samples, the number of alleles per locus (*Na*) ranged from 9 (at locus GAPU71B) to 21 (at locus UDO-043). Heterozygosity metrics further emphasized this genetic diversity. The observed heterozygosity (*Ho*) in the West Bank population was high, ranging from 0.70 (DCA5) to 0.97 (DCA9). The expected heterozygosity (*He*) ranged from 0.70 (DCA5 and GAPU71B) to 0.91 (UDO-043), which is comparable to the *He* bounds of the entire World germplasm (0.64 to 0.91). Moreover, the Polymorphism Information Content (*PIC*) was consistently high for the local samples, spanning from 0.63 (DCA5) to 0.91 (DCA9). These values confirm that despite originating from a geographically restricted area, the ancient West Bank olive trees encompass a degree of genetic polymorphism comparable to a global reference collection.

To evaluate the genetic differentiation and relationships between the West Bank population and 22 other countries, a pairwise *Fst* analysis was conducted (Table 2).

The results were highly significant across all pairwise comparisons (*p* = 0.001, based on 999 permutations). The *Fst* matrix revealed relative genetic affinities among populations. The West Bank shares its closest genetic affinity with Italy, presenting the lowest *Fst* value of 0.040. This is closely followed by affinities with Iran (0.048), Malta (0.049), Syria (0.052), and Greece (0.054). Conversely, genetic differentiation increases markedly when comparing the West Bank to western and North African regions; the highest differentiation was observed against Spain (0.078), Egypt (0.068), and Morocco (0.066). The samples from Türkiye, which is geographically close to the West Bank, were genetically different reaching a high value of *Fst* (0.071). These pairwise metrics represent relative genetic differentiation rather than direct historical migration pathways, as differentiation estimates can be influenced by variations in country sample sizes, reference database cultivar compositions, and shared ancestral lineages. Consequently, the low differentiation observed between the West Bank, Italy, and Iran likely reflects regional genetic affinities or ancient shared ancestry rather than direct, unidirectional dissemination.

### 2.3. Genetic Differentiation of Studied Genotypes

**Figure 4:**
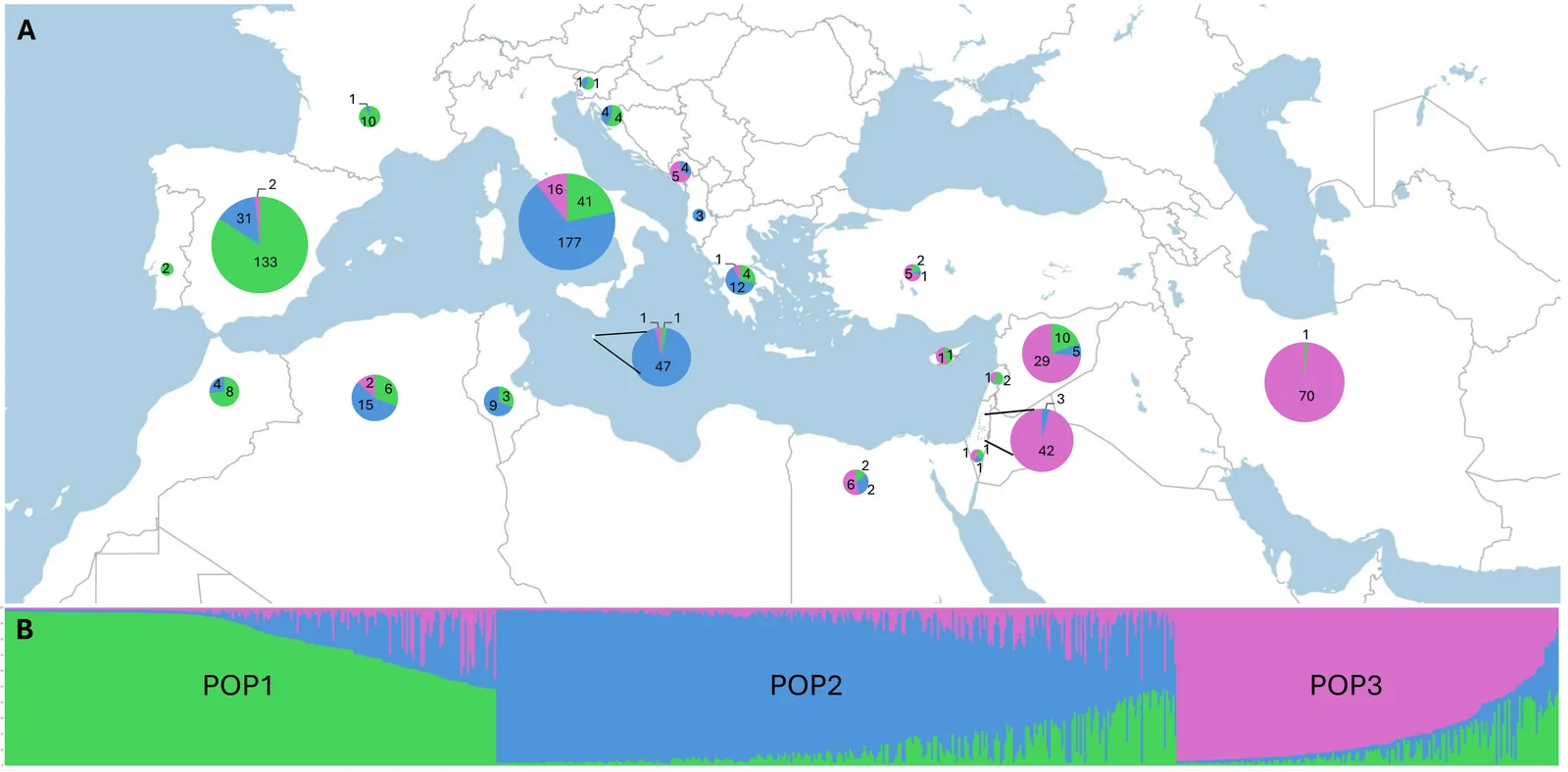
Population genetic structure and geographical distribution of the studied olive accessions. (**A**) Geographical map of the Mediterranean Basin and the Middle East, illustrating the regional distribution of the three genetic populations across the studied countries. The colors inside the circles represent the three genetic populations identified by STRUCTURE, and the numbers inside indicate the number of genotypes assigned to each population. (**B**) Bayesian clustering analysis of the evaluated olive genotypes for K = 3. Each vertical bar represents a single individual, and the distinct colors correspond to the individual’s estimated membership fractions (admixture proportions) assigned to the three inferred genetic clusters: POP1, POP2, and POP3. Detailed country-specific sample sizes and the name of cultivars are provided in Table S2.

To evaluate the genetic architecture of the Palestinian olive trees, the samples were analyzed alongside a worldwide database of 695 genotypes to determine their population structure (Figure 4). To identify the optimal number of genetic clusters (K), the Evanno ΔK method was applied via StructureSelector. This analysis identified K = 3 as the statistically optimal number of clusters, characterized by a sharp, unambiguous absolute maximum peak of ΔK = 12.2 (Figure S3). This selection was further validated by the mean log-likelihood curve [*LnP(K)*], which displayed a steep increase from K = 1 to K = 3 before leveling off into a clear plateau (Figure S3). Based on this Bayesian clustering, the global genetic profiles were divided into three main populations: POP1, POP2, and POP3. The structural analysis reveals that most of the ancient West Bank samples belong almost exclusively to samples collected across diverse Palestinian locations, including Nablus, Tubas, Jenin, and Bethlehem, demonstrate high assignment values to POP3, frequently exceeding 85% to 95%. For instance, rootstocks and canopies from Nablus (e.g., 54R_054NBIZ) and Tubas (e.g., 36R_036TAQA) exhibit POP3 admixture values of 0.972 and 0.966, respectively.

Similarly, samples from Jenin (e.g., 79C_079JQAB) strongly cluster into this population, with values around 0.962. While the POP3 genetic signature dominates the region, a few notable exceptions display different assignments or higher admixture. Specifically, some samples from Hebron diverge from this pattern; for example, sample 84C_084HARR is strongly assigned to POP2 (0.902), and sample 88C_088HARR shows a mixed profile (0.539 in POP2 and 0.310 in POP3).

### 2.4. Parentage Analysis of West Bank Olive Genotypes

To propose statistical parent–offspring hypotheses on breeding networks among monumental olive trees, a parentage analysis was conducted using CERVUS v3.0.7 (Table S3). Pairwise and trio parentage assignments were interpreted as log-likelihood support under a codominant Mendelian inheritance model, validated by manual allele matching, and evaluated within the broader context of the regional population structure. Specifically, the analysis evaluated the roles of the widespread local cultivar ‘Baladi’ and the prominent eastern founder ‘Safrawi’, alongside the unique paleo-genotypes identified in the rootstocks.

### 2.4.1. The Role of ‘Baladi’ and ‘Safrawi’

Direct pairwise parentage analysis revealed that parentage patterns (Table S3). ‘Baladi’ demonstrated a direct genetic match (0 loci mismatching, LOD = 9.65) with the cultivar ‘Abbadi’, together with the Syrian ‘Tarabelsi’ and the Lebanese ‘Souri’, both with only one mismatch and a positive LOD score.

In contrast, ‘Safrawi’ exhibits genetic linkages with the rootstock population. Direct pairwise comparisons identified ‘Safrawi’ as a candidate parent (forming putative parent-offspring relationship) for several unique rootstock genotypes, including 16R_016JBUR, 1R_001JRUM, 21R_021JQAB, 45R_045JMIS, and 55R_055NASI, each with only 1 mismatching locus and positive pair LOD scores ranging from 4.34 to 9.01 (Table S3). ‘Safrawi’ also displayed positive LOD scores with 2 mismatches to numerous other unique local genotypes, such as 14R_014JBUR, 19R_019JQAB, 34R_034TAQA, and 47R_047JMIS.

### 2.4.2. Pedigree Reconstruction and Trio Assignments

Trio parentage analysis was utilized to model the breeding network among the unique ancient genotypes (Table S4). We caution that a positive LOD score alone does not represent definitive proof of direct parentage. Accordingly, the most robust assignment in the dataset (0 loci mismatching, highest Trio LOD = 31.2 and resolving at >95% confidence) supported the statistical hypothesis identifying the unique genotype 55R_055NASI as a putative offspring of a cross between two other unique rootstocks: 26R_026TTAY and 45R_045JMIS.

Further high-confidence trio assignments (allowing for up to 2 mismatches to account for potential somatic mutations or scoring errors) illustrate an extensive web of relationships among these ancient trees (Table S4). Entries in Tables S4 and S5, exhibiting three or four mismatching loci, represent weaker, lower-confidence pedigree hypotheses that are reported for dataset transparency but were excluded from our primary reconstruction of the regional breeding network. The monumental poly-cormic tree 94C_094BWAL, which features a maximum 6.02 m trunk diameter, acts as a critical multi-generational bridge in this ancient network; it was identified as the offspring of 28R_028TTAY and 55R_055NASI (Trio LOD = 12.8). In turn, it successfully reproduced alongside 48R_048JMIS to create another unique rootstock, 31R_031TAQA (Trio LOD = 18.6). Other significant crosses shaping this localized gene pool include 47R_047JMIS (deriving from 32C_032TAQA and 41R_041JMIS; Trio LOD = 24.1), 45R_045JMIS (originating from a cross between 16R_016JBUR and 55R_055NASI; Trio LOD = 19.9), and 32C_032TAQA (identified as the offspring of 31R_031TAQA and 40R_040JSIR; Trio LOD = 14.6).

Finally, to evaluate direct genetic relationships among the ancient Palestinian olive trees, first-degree parentage was analyzed for each individual (Table S5). Twenty-seven genotypes were identified with at least one direct parent, characterized by positive pairwise and top LOD scores, with mismatches ranging from 0 to 2 loci.

## 3. Discussion

### 3.1. Uncovering the Hidden Genetic Reservoir in Ancient Rootstocks

The dual-sampling approach of analyzing both canopy and rootstock tissues independently demonstrates that ancient, living trees function as critical in situ repositories of genetic diversity [7,11]. Previous studies have predominantly relied on canopy sampling, which highlights the intra-varietal diversity of common Levantine landraces such as ‘Nabali Baladi’, ‘Souri’, and ‘Nabali Mohassan’ [8,9], while underestimating the broader genetic richness preserved in underlying root systems. Our findings confirm that the highest genetic variation is hidden within the root systems of these grafted monumental trees.

The high proportion of detected grafting events in the West Bank aligns with findings in other Mediterranean regions, where monumental trees frequently display distinct genetic profiles between their canopy and root-suckers, such as in Israel and the Palestinian Authorities [13], Cyprus [10], and Malta [11]. Grafting has been intimately linked to olive cultivation since antiquity, enabling early agriculturists to clone desirable fruit traits potentially utilizing diverse root systems to adapt to marginal soils, severe drought, and local pathogens, though further physiological experiments are required to confirm these adaptive traits [16,17,18]. The use of ‘Safrawi’ as a rootstock confirms the widespread cultivation of this cultivar, likely predating the ‘Baladi’ cultivar, which previous reports identified as having a foundational role in olive domestication [19]. The rare reverse grafting event (a unique canopy on a ‘Baladi’ rootstock) further illustrates the dynamic and empirical nature of ancient orchard management in the Levant. Similarly, recent investigations into monumental olive trees on Capri Island, some with an estimated age of 932 years after radiocarbon dating, revealed unique genetic profiles that did not match any of the hundreds of worldwide reference varieties [12]. Even singular patriarchal trees, such as the >700-year-old “Olivo della Strega” in Tuscany, have been shown to harbor completely unknown genotypes alongside known varieties like ‘Frantoio’ due to historic grafting [20]. Most of the studied trees have a trunk diameter more than 100 cm, and some even more than 200 cm. Remarkably, all the trees having a trunk diameter exceeding 200 cm represent monumental specimens of presumed multi-centennial age, although precise age estimation in the absence of independent radiocarbon dating remains challenging [21].

### 3.2. The West Bank as a Stronghold of the Eastern Mediterranean Gene Pool

When contextualized globally, the West Bank olive germplasm is overwhelmingly assigned to the POP3 genetic cluster. This highly predominant assignment (characterized by individual membership Q-values frequently exceeding 85% to 95%, such as 0.972 for 54R, 0.966 for 36R, and 0.962 for 79), supports the hypothesis that the Palestinian territories represent a primary stronghold for the Eastern Mediterranean gene pool and aligns these monumental trees with the Levantine primary diversification area [1,2,22]. The POP3 lineage is genetically anchored by ancient eastern founders such as the Safrawi cultivar, which played a pivotal role in the early spread of olives [19].

The three structural populations identified in this global set are consistent with possible regional patterns of differentiation: POP3 represents the Eastern Mediterranean Basin and extensions eastward; POP1 clusters western Mediterranean cultivars around the founder ‘Gordal Sevillana’ (Giarraffa) and represents westward expansion; and POP2 encompasses Central Mediterranean cultivars associated with secondary founders like ‘Canino’ and ‘Frantoio’. The complete absence of POP1 in the West Bank indicates that the core Palestinian orchards remained genetically isolated from westward maritime breeding loops and subsequent commercial selections (e.g., in Andalusia). Unlike central and western Mediterranean populations (POP2 and POP1, respectively), which underwent historical range shifts, extensive selective breeding around secondary founders like Gordal Sevillana or Frantoio, and introgression with native wild oleasters [14,19], the West Bank population remained largely isolated from these later western breeding loops. Our genetic distance clustering analysis clearly demonstrates that the Palestinian rootstocks form their own highly distinct genetic branches, separated by a great genetic distance from standard modern commercial lines (e.g., Arbequina, Koroneiki, Arbosana). This prominent genetic separation, combined with pairwise values that map the closest genetic affinities to Italy (*F_ST_* = 0.040), followed closely by Iran (0.048), Malta (0.049), Syria (0.052), and Greece (0.054), while demonstrating the highest differentiation against Spain (0.078) and Türkiye (0.071) [15], indicates that the West Bank monumental trees represent an ancestral and largely isolated genetic reservoir of the earliest domesticated olives. The isolated deviations or mixed profiles observed in Hebron (such as sample 84 with 90.2% assignment to POP2) could reflect localized introgression, the later introduction of allochthonous commercial varieties, or different historical grafting events compared to the older monumental trees in the northern and central West Bank.

### 3.3. Agronomic Potential and Conservation Implications

The remarkably high heterozygosity and (*Ho* up to 0.97 at locus DCA9 and 0.87 at loci DCA3 and GAPU103A) and allelic richness (131 total alleles across 10 loci, with up to 21 alleles at locus UDO-043) found within this geographically restricted area directly rivals that of extensive World germplasm collections (which contains 257 alleles across 695 international cultivars). Today, global olive cultivation is heavily reliant on a narrow selection of commercial cultivars, making modern orchards increasingly vulnerable to changing climatic conditions and emerging diseases such as *Xylella fastidiosa* [11,12]. The unexploited genetic pool hidden within the ancient Palestinian rootstocks holds considerable agronomic value. These primeval genotypes have survived millennia of challenging environmental fluctuations in the Levant. Reintroducing these locally adapted paleo-varieties into modern breeding programs could provide putative traits for drought tolerance and disease resistance as breeding potential, although further phenotypic or stress-response experiments are required to validate these characteristics [6,7]. Consequently, the in situ conservation of these monumental trees is not only vital for preserving cultural and historical heritage but is also an important measure for the future sustainability of global olive agriculture.

The departures from Hardy–Weinberg equilibrium (HWE) and the negative fixation indices observed among the 48 unique Palestinian genotypes (such as *F* = −0.26 at locus DCA9; Table 1) are typical genetic features of long-lived, outcrossing species. Because olive is characterized by a diallelic self-incompatibility system, sexually reproduced seedlings, which represent the local rootstock pool, typically retain high levels of heterozygosity. Furthermore, these 48 unique genotypes do not represent a single, panmictic breeding population in equilibrium, but rather a cohort of distinct, monumental specimens of presumed old age collected across geographically separated governorates. Finally, in small cohorts (n = 48), minor allele frequency differences between parental pools can mathematically result in an excess of heterozygosity (*Ho* > *He*). Since estimated null allele frequencies at DCA9 are zero (*F_null_* = 0.00), this heterozygosity represents a biological state rather than a scoring artifact. This indicates that heterozygous rootstocks could represent potential genetic features supporting long-term survival under local environmental constraints, reflecting the accumulation of these genotypes over time.

### 3.4. Pedigree Networks and the Distinct Roles of ‘Safrawi’ and ‘Baladi’

The parentage analysis provides empirical data regarding the genetic origins and breeding networks of the monumental olive orchards in the West Bank. The analysis indicates the role of ‘Safrawi’ as a genetic founder within the Levant. Consistent with recent genomic studies identifying ‘Safrawi’ as a founder for modern worldwide olive cultivars [10,19], the present results support likelihood-based parentage hypotheses linking ‘Safrawi’ to several unique Palestinian rootstocks (e.g., 16R_016JBUR, 45R_045JMIS, and 55R_055NASI). This genetic linkage, characterized by high pairwise LOD scores with only 1 mismatching locus (e.g., LOD = 9.01 for 55R_055NASI, LOD = 8.92 for 21R_021JQAB, LOD = 6.75 for 16R_016JBUR, and LOD = 4.34 for 45R_045JMIS), suggests that the rootstocks preserved below ground in the West Bank are descendants or kin of early Eastern genotypes.

These reconstructed pedigree networks reflect a highly dynamic, historically interbreeding population within the Levantine germplasm. Because likelihood-based parentage analysis estimates statistical probabilities under codominant Mendelian inheritance rather than physically tracing linear historical ancestry, these highly probable assignments are best understood as a representation of a highly interconnected regional gene pool. This high genetic connectivity is biologically consistent with the elevated observed heterozygosity and allelic richness characterizing the rootstock population, and contrasts sharply with the absolute clonal uniformity and pedigree isolation of the dominant ‘Baladi’ scion. Nonetheless, the local canopy cultivar ‘Baladi’ is absent from the pedigree network of unique rootstocks. While ‘Baladi’ shares a direct parentage match with the local cultivar ‘Abbadi’ (Syrian cultivar) (0 mismatches, pairwise LOD = 9.65) and a close relationship with ‘Souri’ and ‘Tarabelsi’ (1 mismatch, LOD = 2.35 and 1.47, respectively), its genetic mismatches and negative LOD scores when compared against the rootstocks provide information on historical arboricultural strategies. The genetic separation between the canopies and their respective root systems indicates that ‘Baladi’ did not co-evolve directly from this specific rootstock network. Instead, ‘Baladi’ was propagated via grafting onto a distinct pool of adapted rootstocks, likely selected for its specific above-ground agronomic traits. This practice is consistent with findings from ancient olive trees in other Mediterranean regions, where scions were grafted onto sexually reproduced or feral rootstocks.

Furthermore, the trio parentage reconstructions reveal that the unique rootstock genotypes belong to this interconnected genetic network, providing molecular support for statistical hypotheses of ancestral sexual reproduction rather than vegetative clonal drift. The high-confidence assignments, such as the direct cross between 26R_026TTAY and 45R_045JMIS yielding 55R_055NASI (Trio LOD = 31.2), illustrate multiple generations of localized sexual reproduction. The polycormic tree 94C_094BWAL, which features a trunk diameter of 6.02 m, acts as a generational bridge within the network, serving as both an offspring of older unique genotypes (reconstructed as a cross between 28R_028TTAY and 55R_055NASI; Trio LOD = 12.8, with 2 mismatches) and a parent to subsequent unique rootstocks like 31R_031TAQA (reconstructed as a cross with 48R_048JMIS; Trio LOD = 18.6, with 2 mismatches. This interconnected genetic network suggests that farmers sourced their rootstocks from sexually reproducing populations, utilizing cross-pollinated seedlings as root systems for their grafted clones.

By establishing a robust molecular baseline for Palestinian rootstock diversity, this study opens vital avenues for future research, including high-density SNP profiling, absolute radiocarbon age determination, and functional stress-tolerance screening. Unlocking the genetic architecture and physiological potential of this relict Levantine germplasm will be pivotal for developing climate-smart olive breeding strategies and safeguarding ancient agro-biodiversity.

## 4. Materials and Methods

### 4.1. Plant Material and DNA Extraction

**Figure 5:**
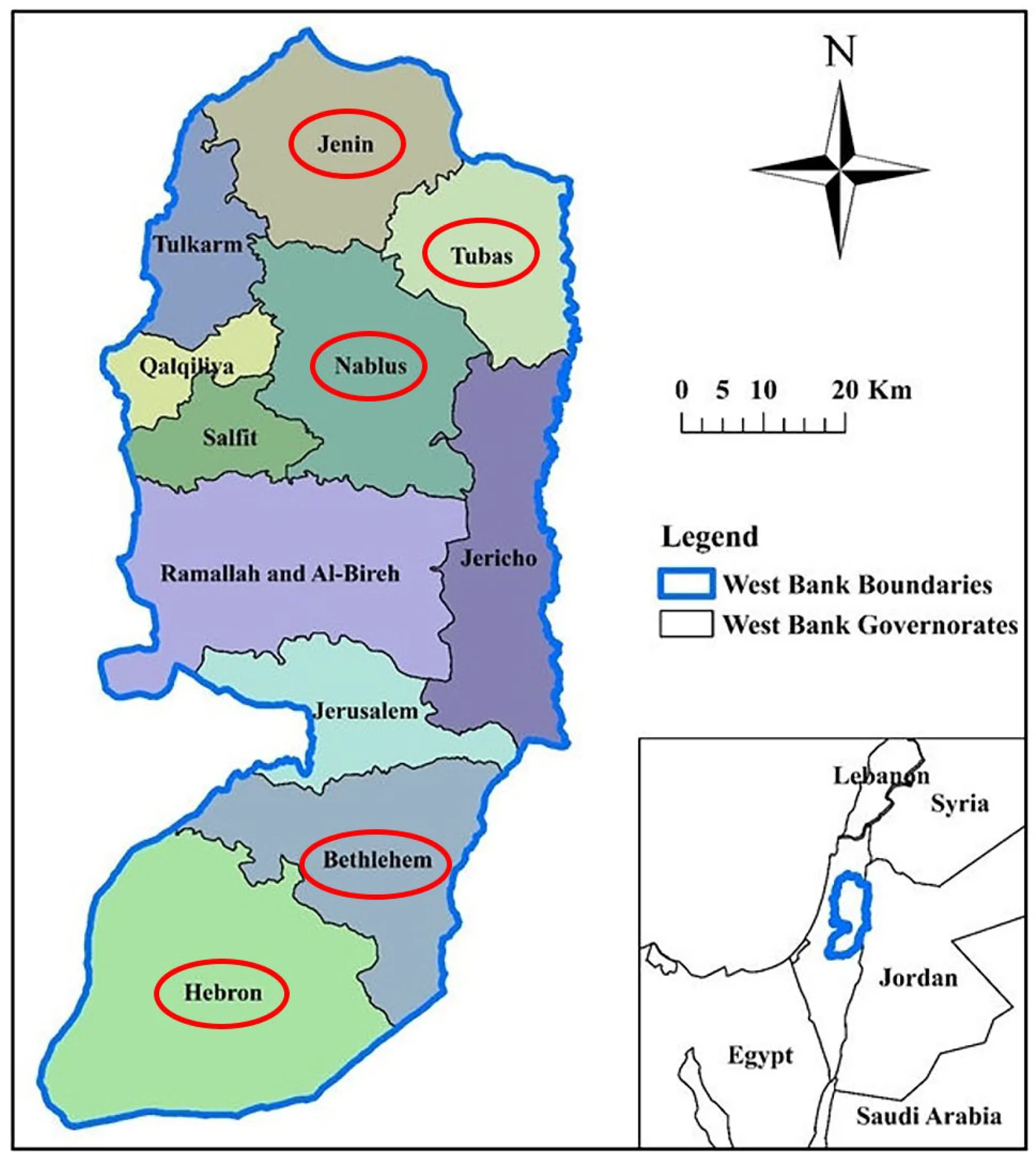
Geographic location of sampled olive trees across the West Bank [25]. Red circles indicate the five sampled governorates: Jenin, Tubas, Nablus, Bethlehem, and Hebron. To ensure traceability, each sampled tree was assigned an alphanumeric code comprising a two-digit ID, governorate initial, and locality tag (e.g., 01JRUM); canopy samples were suffixed “C-” and rootstock subsamples were suffixed “R-” (Table S1).

A total of 93 olive trees were sampled across the West Bank (Jenin, Tubas, Nablus, Hebron, and Bethlehem; Table 3 and Table S1; Figure 5). Ancient trees were prioritized using a >1.0 m trunk diameter at 130 cm from the base of each tree. Because many ancient Mediterranean olives may have been grafted, we collected leaf samples separately from canopy and rootstock whenever accessible. To ensure precise identification of the rootstock samples, leaves were collected exclusively from young basal suckers emerging directly from the trunk below the visible graft union or from active shoots arising from the shallow root system. In total, 142 leaf and sucker samples were collected, representing 54 ancient monumental trees (>100 yrs, >1.0 m) and 39 (<100 yrs, <1.0 m) reference local cultivar accessions. Sampling comprised 49 paired trees (both canopy and rootstock sampled), 43 canopy-only trees, and 1 rootstock-only tree, categorized across ancient monumental specimens (>1.0 m trunk diameter) and young reference trees. A comprehensive summary of the sampling design and sample counts across tree category is presented in Table 3. Samples were transported chilled and stored at 4 °C until DNA extraction.

### 4.2. Nuclear Marker Applied to Olive Genotypes

To determine and verify the identity of the accessions, all samples were genotyped using standard dinucleotide SSR markers, which are widely utilized for cultivar characterization in olive germplasm collections [15,23,24]. Ten highly polymorphic markers, previously identified as the best-performing loci, were selected: DCA3, DCA5, DCA9, DCA16, DCA18, EMO-90, GAPU71B, GAPU101, GAPU103A, and UDO99-043. Forwardprimers were labeled at the 5′ end with FAM, VIC, PET, or NED fluorophores. The chromosomal positions for each SSR marker are provided in Valeri et al. [11]. The complete multilocus SSR genotype matrix containing the harmonized allele calls across all 10 SSR loci for all 743 analyzed samples (comprising the 48 unique West Bank genotypes and 695 international reference cultivars) is provided in full in Table S6.

Genomic DNA was extracted from young leaves using a CTAB protocol optimized for polyphenol-rich tissues, followed by chloroform: isoamyl alcohol clean-up, alcohol precipitation, and resuspension in nuclease-free water [26]. DNA quantity and purity were assessed spectrophotometrically (A260/280 ≈ 1.8–2.0) using a NanoPhotometer^®^ NP80 (Implen GmbH, Munich, Germany). PCR amplifications were conducted according to the method described by [15]. To visualize the alleles’ peaks, fluorescently labeled fragments were resolved via capillary electrophoresis on a SeqStudio Genetic Analyzer (Thermo Fisher Scientific, Waltham, MA, USA) using the GeneScan™ 500 LIZ internal size standard (Thermo Fisher Scientific, USA). Data analysis was performed using GeneMapper™ Software v.6 (Thermo Fisher Scientific, USA). Allele binning was performed by systematically standardizing raw fractional fragment lengths obtained from capillary electrophoresis (e.g., 124.3 bp) into discrete integer allele sizes (e.g., 124 bp). This standardization ensures that genetic profiles can be accurately grouped and compiled into the final genotypic distance matrix (Table S6). Subsequently, strict cultivar matching criteria were applied: genotypes were classified as identical and assigned to a known reference cultivar or a specific clonal group, only if they exhibited a perfect allelic match across all 10 evaluated SSR loci.

### 4.3. Frequency Analysis, Genetic Distance Dendrogram and Bayesian Analysis

Genotypes with unique profiles, excluding genotypes identical to the known cultivars, were selected for frequency analysis. The following population-level genetic statistics were calculated using GenAlEx 6.5 [27]: number of alleles (*Na*), number of effective alleles (*Ne*), observed heterozygosity (*Ho*), expected heterozygosity (*He*), and fixation indices (*F*). To determine the genetic uniqueness of each accession and quantify redundancy, the polymorphic information content (*PIC*) and the frequency of potential null alleles (*F_null_*) were calculated for each microsatellite locus using CERVUS v.3.0.7 software [28]. To identify the genetic uniqueness of each accession, estimate the frequency of potential null alleles (*F_null_*), and perform parentage analysis, CERVUS v3.0.7 software was utilized. The allele frequency analysis was conducted on the total dataset of 743 genotypes, with the Hardy–Weinberg test calculated using a minimum expected frequency of 5, applying Yates’ correction when degrees of freedom (*df*) = 1, and implementing the Bonferroni correction to assess statistical significance. For parentage simulation, the parameters were defined as follows: parent pair sexes were set as unknown (as the olive tree is a hermaphroditic species); 10,000 offspring were simulated; the proportion of candidate parents sampled was set to 5%; the proportion of loci typed was set to 20%; the proportion of loci mistyped (genotyping error rate) was set to 5%; and the minimum typed loci threshold was set to 8. Statistical confidence was calculated using critical delta (Δ) values derived from LOD scores at a relaxed threshold of 80% and a strict threshold of 95%. Pairwise and trio parentage analyses were performed by searching for the two most-likely parents for each offspring, ranking them by their joint LOD score and calculating non-exclusion probabilities. Genotypes of the offspring themselves were included among the candidate parents to account for potential self-pollination. Finally, all parentage assignments generated by the software were manually verified by comparing the allele sizes of each offspring and its proposed parent(s) to guarantee biological compatibility. To ensure maximum statistical confidence, assignments were formally classified as ‘high-confidence’ only when they resolved at the strict 95% simulation-derived confidence threshold and exhibited ≤2 mismatching loci; assignments with more than two mismatching loci or resolving at the 80% relaxed threshold were treated strictly as putative, lower-confidence parentage hypotheses rather than definitive pedigrees.

Furthermore, GenAlEx 6.5 was employed to estimate a pairwise *Fst* analysis to differentiate the studied regional genotypes from worldwide cultivars.

The SSR data from the 48 Palestinian genotypes in the current study were compared with a representative sample of 695 cultivars from across the Mediterranean and beyond, here representing the ‘World’ population, resulting in a total combined dataset of 743 genotypes (Tables S1, S2 and S6). To ensure compatibility between the newly generated profiles and the reference database, all genotypes were evaluated using the identical set of 10 SSR loci and standard PCR amplification conditions. Peak sizing and allele binning were standardized across capillary electrophoresis runs by using standard reference cultivars (including ‘Frantoio’, ‘Leccino’, ‘Picual’, and ‘Arbequina’) as internal sizing controls, ensuring the direct comparability of the final allele calls. To infer genetic relationships, a phylogenetic tree was constructed in MEGA7 [29] using the neighbor-joining (NJ) method.

Genetic structure was further analyzed using STRUCTURE 2.3.4 software [30]. The analysis utilized an admixture model with independent allele frequencies, running 100 iterations for each K value (ranging from K = 1 to 7). Each run consisted of a 100,000-step burn-in period followed by 50,000 Markov Chain Monte Carlo (MCMC) repetitions. The most likely value of K was subsequently estimated using StructureSelector [31], according to the Evanno ΔK method [32], which identifies the uppermost hierarchical level of genetic structure based on the second-order rate of change of the likelihood function with respect to K. To reconcile label switching and multi-modality across independent runs, the 100 replicate runs for each K were aligned and averaged using the CLUMPAK pipeline (incorporating the CLUMPP Greedy algorithm [33] with 1000 random input order permutations) to generate the final, consensus membership coefficient (Q) matrix. Run convergence was formally evaluated by assessing the standard deviation of the log probability values [*LnP(K)*] across iterations and by confirming that the pairwise similarity coefficient (*H′*) between the 100 runs exceeded 0.99 for the selected K value.

## 5. Conclusions

By independently genotyping the canopy and rootstock tissues of monumental olive trees in the West Bank, this study provides molecular evidence of historical grafting practices. Early agriculturists combined uniform scions, predominantly the local ‘Baladi’ cultivar, with a highly diverse pool of uncharacterized rootstocks. This dual-sampling approach reveals a substantial genetic reservoir preserved below ground. Parentage analysis further clarifies the genetic history of these orchards, proposing ‘Safrawi’ as a foundational candidate parent that putatively contributed to this diverse rootstock population and revealing interconnected pedigree networks. Likelihood models suggest that these unique rootstocks form part of a historically interbreeding population derived from multi-generational seedling crosses, rather than being isolated feral mutations. Furthermore, the genetic separation of ‘Baladi’ from this rootstock pedigree indicates its distinct selection for specific above-ground traits and its subsequent propagation via grafting onto diverse root systems. The assignment of the West Bank samples to the POP3 genetic cluster suggests that the Palestinian territories are a major historical stronghold for the Eastern Mediterranean germplasm. Remaining largely isolated from the hybridization and breeding loops that shaped Western Mediterranean commercial cultivars, the West Bank trees have retained the ancestral genetic signatures of the early Eastern founders. Faced with global climate change and emerging pathogens, the genotypes hidden within these monumental rootstocks putatively possess considerable agronomic potential. Consequently, their in situ conservation will provide a critical genetic source for future analyses on drought tolerance, disease resistance, and physiological stress adaptation in climate-resilient olive agriculture.

## Figures and Tables

**Table 1:** Indices of genetic diversity of 743 samples for each SSR locus: number of alleles (*Na*). number of effective alleles (*Ne*). Shannon’s information index (*I*), observed heterozygosity (*Ho*), expected heterozygosity (*He*), fixation index (*F*), Polymorphism Information Content (*PIC*), deviation from Hardy–Weinberg equilibrium and Frequency of null alleles were reported for each analysed locus.

Population	Locus	*Na*	*Ne*	*I*	*Ho*	*He*	*F*	*PIC*	*HW*	*F(Null)*
West Bank	DCA3	17.00	8.04	2.39	0.87	0.87	0.00	0.87	***	−0.01
	DCA5	10.00	3.41	1.71	0.70	0.70	−0.00	0.63	**	0.05
	DCA9	13.00	4.40	1.86	0.97	0.77	−0.26	0.91	***	0.00
	DCA16	12.00	4.39	1.78	0.79	0.77	−0.02	0.89	***	0.01
	DCA18	11.00	4.19	1.74	0.83	0.76	−0.09	0.83	***	−0.02
	EMO90	11.00	4.07	1.71	0.77	0.75	−0.02	0.69	***	0.01
	GAPU71B	9.00	3.40	1.52	0.77	0.70	−0.09	0.79	***	−0.01
	GAPU101	12.00	4.52	1.82	0.79	0.77	−0.01	0.86	***	−0.01
	GAPU103A	15.00	5.20	2.06	0.87	0.80	−0.08	0.87	***	0.08
	UDO-043	21.00	11.84	2.68	0.79	0.91	0.13	0.90	***	0.04
World germplasm	DCA3	28.00	8.82	2.41	0.90	0.88	−0.02	0.86	ND	0.00
	DCA5	17.00	2.82	1.63	0.58	0.64	0.09	0.68	NS	0.00
	DCA9	28.00	12.38	2.80	0.91	0.91	0.00	0.74	*	−0.13
	DCA16	44.00	10.40	2.68	0.86	0.90	0.03	0.74	NS	−0.01
	DCA18	21.00	6.57	2.14	0.88	0.84	−0.03	0.73	NS	−0.06
	EMO90	16.00	3.60	1.68	0.69	0.72	0.04	0.72	NS	−0.0
	GAPU71B	20.00	5.39	1.91	0.83	0.81	−0.02	0.66	NS	−0.04
	GAPU101	19.00	8.22	2.26	0.90	0.87	−0.03	0.74	NS	−0.01
	GAPU103A	34.00	8.95	2.56	0.74	0.88	0.16	0.78	NS	−0.04
	UDO-043	30.00	10.71	2.65	0.82	0.90	0.08	0.90	ND	0.07

Asterisks indicate *** *p* < 0.001, ** *p* < 0.01 and * *p* < 0.05 and NS represents not significant values.

**Table 2:** Pairwise analysis *Fst* among the 12 populations represented by the largest number of samples.

Population 1	Population 2	*Fst*	*p*(Rand ≥ Data)
West Bank	Italy	0.040	0.001
West Bank	Iran	0.048	0.001
West Bank	Malta	0.049	0.001
West Bank	Syria	0.052	0.001
West Bank	Greece	0.054	0.001
West Bank	Tunisia	0.059	0.001
West Bank	Algeria	0.061	0.001
West Bank	Morocco	0.066	0.001
West Bank	Egypt	0.068	0.001
West Bank	Türkiye	0.071	0.001
West Bank	Spain	0.078	0.001

Probability. *p*(rand ≥ data) based on 999 permutations.

**Table 3:** Schematic summary of the olive sampling design across the West Bank.

Tree Category	Paired Trees (C + R)	Unpaired Canopy (C Only)	Unpaired Rootstock (R Only)	Total Trees	Canopy Samples (C)	Rootstock Samples (R)	Total Leaf Samples
Monumental (>100 yrs, >1.0 m)	45	8	1	54	45	1	91
Young Reference (<100 yrs, <1.0 m)	4	35	0	39	39	4	43
TOTALS	49	43	1	93	92	50	142

## Data Availability

The original contributions presented in the study are included in the article/Supplementary Material. Further inquiries can be directed to the corresponding author.

## Supplementary Materials

The following supporting information can be downloaded at https://www.mdpi.com/article/10.3390/plants15182838/s1. Figure S1. Genetic distance clustering and relationships of the unique genotypes studied in comparison with 695 international cultivars. Red circles indicated the 48 Palestinian unique genotypes (a separate PDF file). Figure S2. Principal Coordinates Analysis (PCoA) of 743 olive genotypes based on 10 SSR markers. Figure S3. Graphical STRUCTURE diagnostics based on the Evanno method. The left panel displays the calculated K values across the tested number of clusters (K), demonstrating that the absolute maximum peak occurs at K = 3, which indicates the statistically optimal hierarchical structure. The right panel illustrates the mean log probability of the data [Mean LnP(K)] across the tested K values, with error bars representing the standard deviation (variance) among the 100 replicate runs. Table S1. Collected samples information. Table S2. International cultivars which have been used in this study to compare with Palestinian genotypes. Table S3. Direct pairwise parentage analysis and LOD scores for “Safrawi” and “Baladi” cultivars evaluated against unique Palestinian olive genotypes. Table S4. Trio parentage analysis and LOD scores for the pedigree reconstruction of unique ancient Palestinian olive genotypes. Table S5. Direct pairwise parentage analysis and positive Pair top LOD scores for West Bank population. Table S6. Pairwise codominant genotypic distance matrix for 48 Palestinian unique genotypes and 695 worldwide cultivars based on 10 SSR loci.
