# Supplementary material for: Uncovering Hidden Genetic Diversity in Ancient Palestinian Olive Rootstocks and Insights into Levantine Domestication
Source: Plants (Basel). 2026 Sep 16;15(18):2838. doi: 10.3390/plants15182838 (PMC13611641; doi:10.3390/plants15182838)

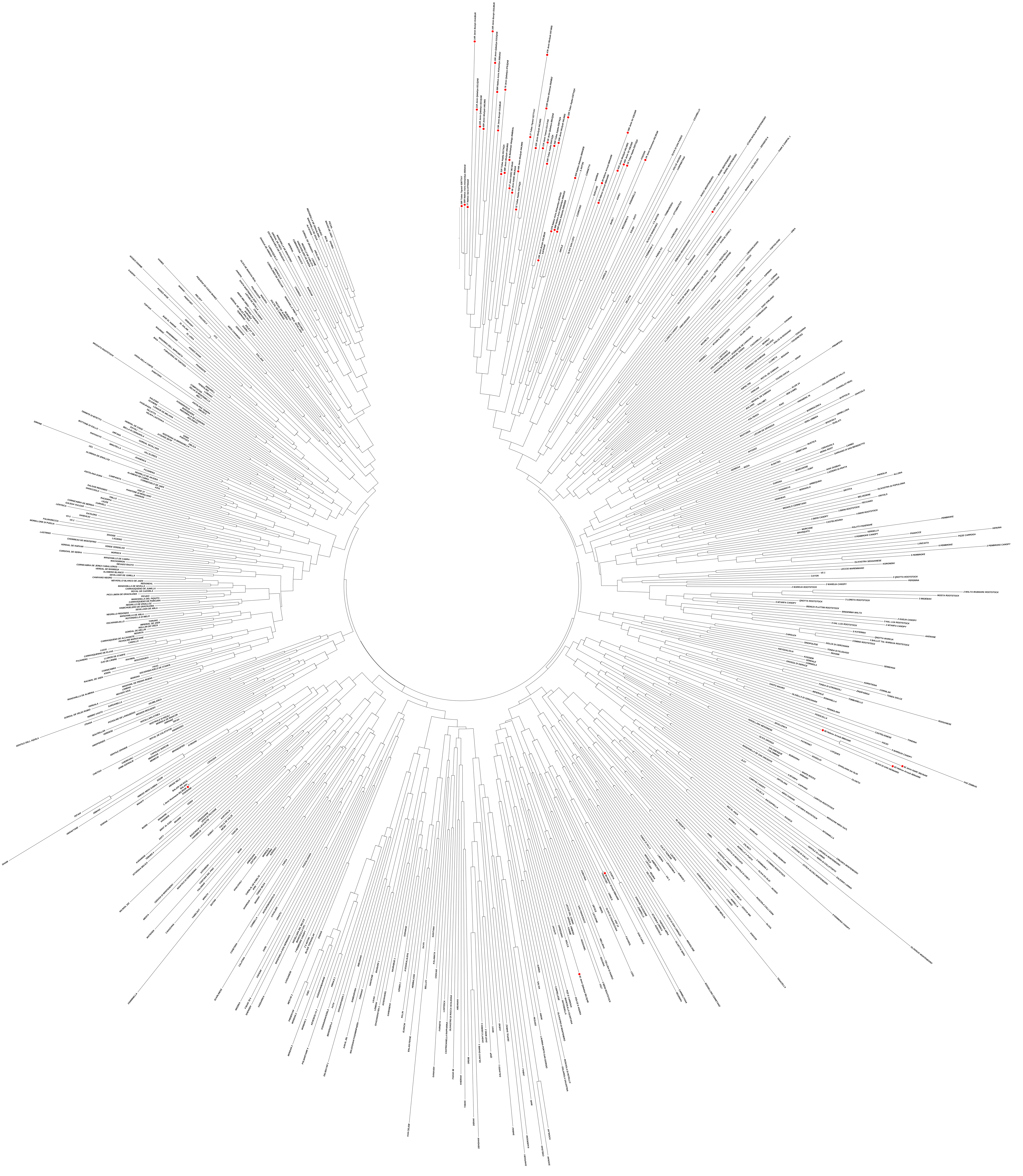

Supplement: Supplementary file 1 [file plants-15-02838-s001.zip › Figure_S1.pdf]
