# Supplementary material for: Uncovering Hidden Genetic Diversity in Ancient Palestinian Olive Rootstocks and Insights into Levantine Domestication
Source: Plants (Basel). 2026 Sep 16;15(18):2838. doi: 10.3390/plants15182838 (PMC13611641; doi:10.3390/plants15182838)
Supplement: Supplementary file 1 [file plants-15-02838-s001.zip › Supplementary_Files.pdf]

Uncovering Hidden Genetic Diversity in Ancient Palestinian Olive Rootstocks and Insights into Levantine Domestication

Ahmad Alomary[1], Roberto Mariotti[2], Osama Alabdallah[3], Lorenzo Cruciani[2++], Muhammad Ajmal Bashir[2], Mohammed M. Abueid[3], Raed Alkowni[4], Silvia Casciani[2], Soraya Mousavi[2*]

Figure S1. Genetic distance clustering and relationships of the unique genotypes studied in comparison with 695 international cultivars. Red circles indicated the 48 Palestinian unique genotypes (a separate PDF file).

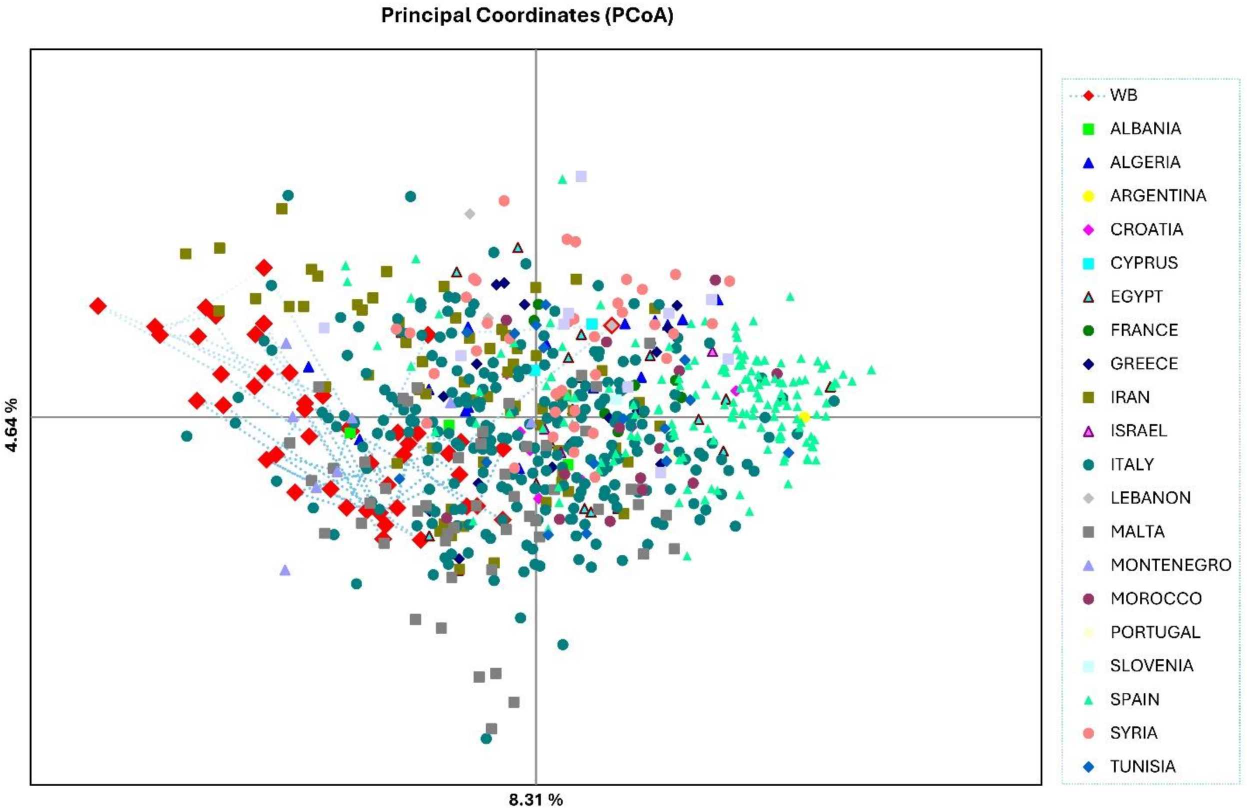

Figure S2. Principal Coordinates Analysis (PCoA) of 743 olive genotypes based on 10 SSR markers.

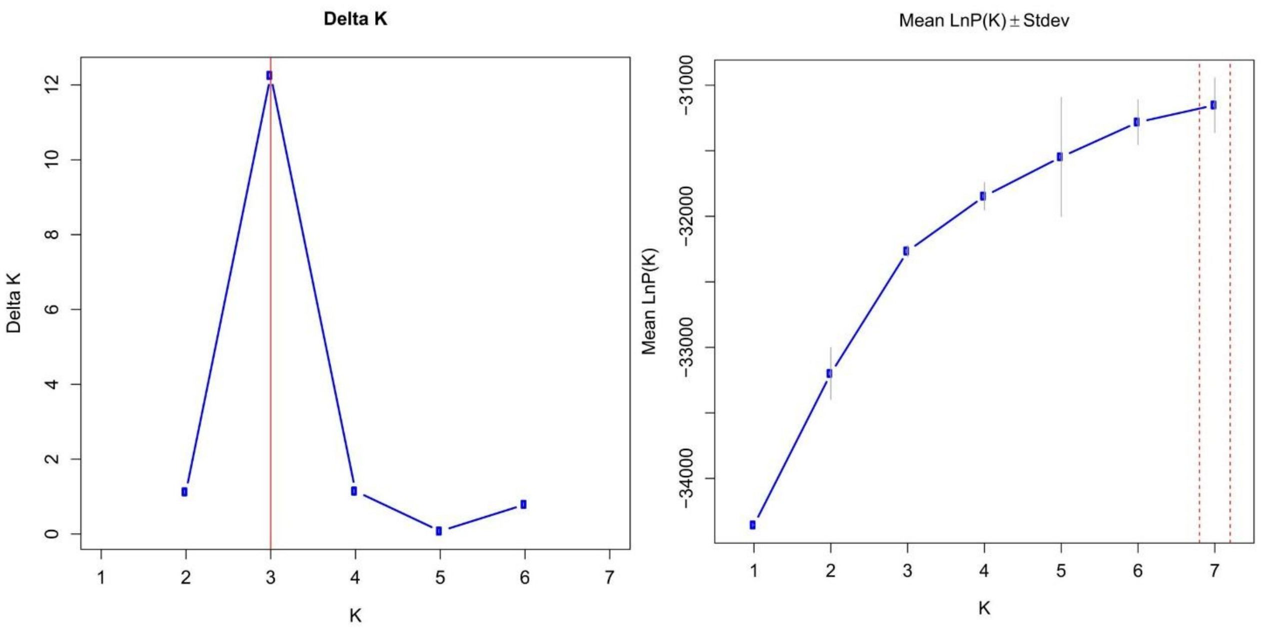

Figure S3. Graphical STRUCTURE diagnostics based on the Evanno method. The left panel displays the calculated K values across the tested number of clusters (K), demonstrating that the absolute maximum peak occurs at K = 3, which indicates the statistically optimal hierarchical structure. The right panel illustrates the mean log probability of the data [Mean LnP(K)] across the tested K values, with error bars representing the standard deviation (variance) among the 100 replicate runs.

Table S1. Collected samples information.

Table S2. International cultivars which have been used in this study to compare with Palestinian genotypes.

Table S3. Direct pairwise parentage analysis and LOD scores for 'Safrawi' and 'Baladi' cultivars evaluated against unique Palestinian olive genotypes.

Table S4. Trio parentage analysis and LOD scores for the pedigree reconstruction of unique ancient Palestinian olive genotypes.

Table S5. Direct pairwise parentage analysis and positive Pair top LOD scores for West Bank population.

Table S6. Pairwise codominant genotypic distance matrix for 48 Palestinian unique genotypes and 695 worldwide cultivars based on 10 SSR loci.
